# Therapeutic Targeting of Nrf2 Signaling by Maggot Extracts Ameliorates Inflammation-Associated Intestinal Fibrosis in Chronic DSS-Induced Colitis

**DOI:** 10.3389/fimmu.2021.670159

**Published:** 2021-08-12

**Authors:** Rong Wang, Daojuan Wang, Hongwei Wang, Tingyu Wang, Yajing Weng, Yaling Zhang, Yongzheng Luo, Yadong Lu, Yong Wang

**Affiliations:** ^1^State Key Laboratory of Analytacal Chemistry for Life Science & Jiangsu Key Laboratory of Molecular Medicine, Medical School, Nanjing University, Nanjing, China; ^2^ School of Chemistry and Life Sciences, Jinling College, Nanjing University, Nanjing, China; ^3^Neonatal Medical Center, Children’s Hospital of Nanjing Medical University, Nanjing, China

**Keywords:** IBD, inflammation, intestinal fibrosis, Nrf2, TGF-β1/Smads pathway, maggot extracts

## Abstract

Intestinal fibrosis is induced by excessive myofibroblast proliferation and collagen deposition, which has been regarded as a general pathological feature in inflammatory bowel disease (IBD). Therefore, identifying clinical markers and targets to treat and prevent intestinal fibrosis is urgently needed. The traditional Chinese medicine maggot, commonly known as “wu gu chong”, has been shown to reduce oxidative stress and alleviate inflammation in chronic colitis. This study investigated the mechanisms underlying the effects of maggot extract (ME) on inflammation-associated intestinal fibrosis in TGF-β1-stimulated human intestinal fibroblasts (CCD-18Co cells) and dextran sodium sulphate (DSS)-induced chronic colitis murine model. To assess the severity of inflammation and fibrosis, histological and macroscopic evaluation were carried out. The results showed that ME was a significant inhibitor of body weight loss and colon length shortening in mice with chronic colitis. In addition, ME suppressed the intestinal fibrosis by downregulating TGF-β1/SMADs pathway *via* upregulation of Nrf2 expression at both protein and mRNA levels. ME markedly increased the expression of Nrf2, thus resulting in a higher level of HO-1. After treatment with Nrf2 inhibitor (ML385) or siRNA-Nrf2 for deactivating Nrf2 pathway, the protective effects of ME were abolished both *in vitro* and *in vivo*. Moreover, the histopathological results for the major organs of DSS mice treated with ME showed no signs of clinically important abnormalities. Treatment with ME had no effect on the viability of CCD-18Co cells, suggesting its low *in vitro* cytotoxicity. Furthermore, ME could mediate intestine health by keeping the balance of the gut microbes through the enhancement of beneficial microbes and suppression of pathogenic microbes. In conclusion, this is the first ever report demonstrating that ME ameliorates inflammation-associated intestinal fibrosis by suppressing TGF-β1/SMAD pathway *via* upregulation of Nrf2 expression. Our findings highlight the potential of Nrf2 as an effective therapeutic target for alleviating intestinal fibrosis.

## Introduction

Inflammatory bowel disease (IBD) is one of the chronic relapsing disorders that covers ulcerative colitis (UC) and Crohn’s disease (CD). The development of IBD involves complex interactions between genetic variation, environmental factors, gut microbiota and the immune system. However, the exact molecular pathways and underlying mechanisms remain largely unknown (1, 2). Intestinal fibrosis is a common and incurable complication of IBD, which accounts for 30% of CD patients (3). Due to fibrotic complications, one-third of CD patients require surgical operation (4). However, surgical operation does not confer any protection against disease recurrence and fibrotic complications, which in turn requires additional surgeries. In addition, UC is characterized by long-term complications such as intestinal fibrosis (5). The mechanism of intestinal fibrosis is often complicated and featured with the uncontrolled proliferation of mesenchymal cells and excessive accumulation of extracellular matrix (ECM) proteins (6, 7). Moreover, intestinal fibrosis may cause frequent luminal stenosis with considerable effects on patients’ quality of life, and such vicious circle can lead to disfiguring surgery (8, 9). Given that colon fibrosis and intestinal inflammation often occur together in IBD patients, it is believed that fibrosis is caused by the chronic inflammation of colon cells and delayed colonic healing (7). The injured colon tissue is recovered through the healing program of the intestine under normal conditions; but if the damage is uncontrolled, it may lead to chronic colon inflammation, together with permanent injury and continuous repair cycle, and eventually intestinal fibrosis (10, 11).

Increasing evidence has suggested that insects contain a potential source of bioactive compounds for medicinal uses (12). One of these is maggot, or known as “wu gu chong” in Chinese, which is the larva of *Lucilia sericata*. The maggot lives in a harsh environment in the wild, but it can still adapt well. Thus, we hypothesize that it has a good immune defense mechanism in the body. There are clinical records from the Ming Dynasty (1368 A.D) regarding the use of housefly maggots to treat patients with bacterial infection, ecthyma and wounds in the digestive system (13, 14). The maggot extract (ME) shows positive impact on gastric cancer and coma when used in concomitant with other drugs (13). Therapeutic intervention based on ME has notable antimicrobial activity, and is the most rapid and efficient way to treat wound debridement with necrotic and/or infected tissue (15). It has been reported that ME and maggot secretion (MS) can suppress multiple pro-inflammatory responses, such as chemotaxis, degranulation, integrin expression and respiratory burst, through neutrophil production. However, it is worth noting that ME and MS have no significant effect on the antimicrobial activities of neutrophils (16).

According to our previous experiments, ME can significantly upregulate the expression levels of NF-E2-related factor 2 (Nrf2) (17). As the most dominant nuclear transcription factor, Nrf2 plays a major role in mediating cellular defense against oxidative stress and inflammation (18, 19). Under unstressed conditions, Nrf2 binds with Kelch-like ECH-associating protein 1 (Keap1, a negative regulatory protein), and remains active in the cytoplasm. Upon exposure to oxidative stress or inflammation, Nrf2 dissociates from Keap1 and translocates into the nucleus (20). Nrf2 is then combined with antioxidant response elements to induce the expression of different antioxidant-related genes such as glutamate-cysteine ligase catalytic subunit, home oxygenase-1 (HO-1) and glutamate-cysteine ligase modifier subunit. Through this, Nrf2 can scavenge reactive oxygen species (ROS) and lower the accumulation of oxidative stress substances (21). Previous studies have shown that ROS can cause fibrosis *via* modulation of TGF-β1/SMADs pathway (22, 23). TGF-β1 is one of the main pro-fibrogenic regulators found during intestinal fibrosis, which is regulated through the SMADs family to trigger the release of collagen and fibronectin (the major components of ECM) (24). Besides, the matrix metalloproteinases (MMPs) and tissue inhibitors of metalloproteinases (TIMPs) have been shown to mediate the degradation of ECM. It has been reported that excessive accumulation of ECM is induced by the abnormal expression of MMPs and TIMP-1 (8). Interestingly, TGF-β1 can increase TIMP-1 expression and suppress MMP expression, thereby preventing the degradation of ECM proteins (25). These findings imply that Nrf2 is required to control intestinal fibrosis.

The mechanisms underlying the therapeutic effects of ME on inflammation-associated intestinal fibrosis were elucidated in this study. Our results demonstrated that ME ameliorated inflammation-associated intestinal fibrosis with TGF-β1/SMADs pathway inhibition through overexpressing Nrf2 in chronic DSS colitis and human intestinal fibroblasts.

## Materials and Methods

### Maggots and ME Preparation

Blowflies were housed in cages under normal conditions, and fed with dry diet (sugar/milk powder/yeast; 2/2/1) and milk. The larvae were collected and placed into a bigger pond containing wheat husks, yeast extracts and milk powder. After breeding, the maggots were rinsed with distilled water for three times, followed by heat‐drying. grinding into powder form and mixing with two volumes of phosphate-buffered saline (PBS). ME homogenate was prepared, and then centrifuged for 10 min at 15000 r/min. The collected supernatant was heated in a water bath at 70°C for 30 s before being centrifuged for 10 min at 15000 r/min. Finally, a 0.22-μm mesh was used to filter the resulting supernatant (17).

## Mice and Induction of Colitis by DSS

DSS (MW: 36,000−50,000) was procured from MP Biomedicals (Santa Ana, CA, USA). The Nrf2 inhibitor ML385 was supplied by Selleckchem (Houston, TX, USA). C57BL/6 mice (6-8 weeks old, female, 20 g) were supplied by Model Animal Research Center of Nanjing University. A specific-pathogen-free (SPF) environment (temperature, 22 ± 1°C; relative humidity, 50 ± 1%; and normal day/night cycle, 12/12 h) was set up. The animals were randomly allocated to six groups (n=8 per group): (i) normal control group (sterile water), (ii) chronic colitis group (DSS), (iii) mesalazine group (mesalazine + DSS), (iv) treatment group (ME + DSS), (v) Nrf2 inhibitor group (DSS + ML385 + ME), and (vi) ME group (sterile water + ME).

**Figure 1:**
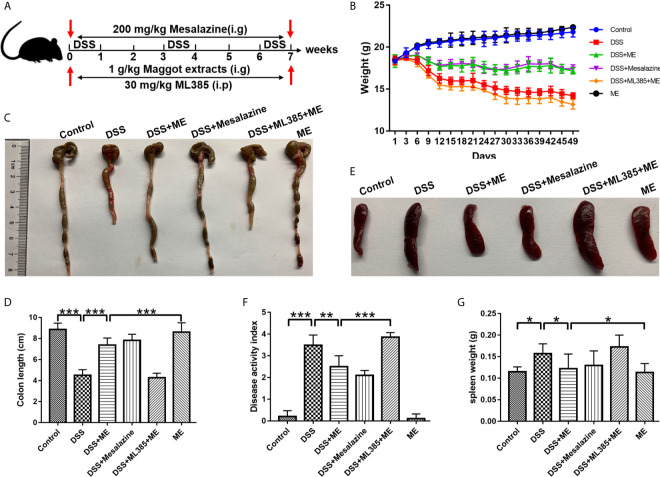
Maggot extract (ME) alleviates the symptoms of chronic colitis in DSS-exposed mice. Mice were allocated to six groups (n=8 per group). **(A)** The mice were exposed to repeated “cycles” of DSS exposure. Every cycle consisting of 2% DSS for seven days and sterile water for fourteen days. In total, the mice were exposed to one, two or three cycles. Subsequently, the mice received an intragastric administration of vehicle control, 1 g/kg ME, 200 mg/kg mesalazine or 30 mg/kg ML385. Body weight **(B)**, macroscopic features **(C, E)**, colon length **(D)**, disease activity index **(F)**, and spleen weight **(G)** were obtained and presented as mean ± SD. *P < 0.05, **P < 0.01, ***P < 0.001.

To induce chronic colitis, the drinking water was mixed with 2% DSS and fed to the acute colitis mice for seven days. To examine DSS-induced chronic colitis, the animals were repeatedly exposed to multiple cycles of DSS. In brief, each cycle contained 2% DSS for 7 days, followed by sterile water for the next 14 days. The subjects received one, two, or three cycles. Day 1 was the first day, while Day 49 was the last day (**Figure 1A**). Control mice received normal drinking water. For treatment group, the mice received 1 g/kg per day (w/w) of ME using an intragastric cannula from the first day of DSS treatment. Otherwise, the subjects received 200 mg/kg (w/w) of mesalazine (the standard prescription drug for IBD), while ML385 (30 mg/kg) pretreatment was intraperitoneally given one hour prior to ME administration. The whole intervention process was done within the same timeframe; therefore, only one control group was needed.

All animals survived the DSS experimental protocol. The daily collected data included the behavior of subjects, body weight, and frequencies of bowel movements. After repeated “cycles”, the colon tissue and serum specimens were collected from the sacrificed animals. The Institutional Animal Care and Use Committee of Nanjing University approved the animal care and *in vivo* experimental procedures based on the guidelines of institutional animal ethics (2019-124210-225A).

### Evaluation of Body Weight, Colon Histopathology, Colon Length and Disease Activity Index (DAI) Score

The severity of DSS-induced chronic colitis in mice was analyzed by DAI scores based on the criteria mentioned earlier. In brief, the loss in body weight, changes in stool consistency, and rectal bleeding were measured. There were four classes of weight losses, including 0 (<5%), 1 (5–10%), 3 (10–20%) and 4 (>20%). In the case of stool consistency, there were normal well-formed particles (0), loose stools (1), semi-formed stools (2), liquid stools (3) and diarrhea (4). In addition, there were five rectal bleeding classes, namely, no blood (0), trace (1), mild hemoccult (2), obvious hemoccult (3) and gross bleeding (4). After that, all the sub-scores were added and the total score was divided by three to yield the DAI scores (ranging from 0 to 4).

The animals were sacrificed through cervical dissociation, and the length of their large intestines was measured. Around 10% of the distal colon was fixed in buffered formalin (10%) and then embedded in paraffin, followed by hematoxylin and eosin (H&E) staining to measure the level of inflammatory reaction. In addition, Masson’s trichrome staining was carried out to measure tissue fibrosis. To assess the levels of tissue damage caused by DSS-induced chronic colitis, histological scores were assessed through ulceration, where no ulcers was scored as 0, small ulcers <3 mm as 1, and large ulcers >3 mm as 2. In addition, no inflammation was scored as 0, mild inflammation as 1, moderate inflammation as 2, and severe inflammation as 3. No depth of lesion was scored as 0, submucosa as 1, muscularis propria as 2, and serosa as 3. No fibrosis was scored as 0, mild fibrosis as 1, and severe fibrosis as 2. The remaining colon specimens were stored at -80°C for RNA and protein extraction.

### Cell Culture Conditions andDrug Treatment

Human intestinal CCD-18Co fibroblasts cell lines and RAW 264.7 murine macrophage cell lines were supplied by Heyi (Suzhou Heyi Biotech Co. Ltd., Suzhou, China). Dulbecco’s modified Eagle medium (DMEM) with high glucose (Life Technology, NY, USA) was used to culture the cells. The medium was added with 100 mg/ml streptomycin, 100 units/ml penicillin and 10% fetal bovine serum (Hyclone, Logan, UT, USA). The cells were maintained at 5% CO_2_, humidity ≥90%, 37 °C. The Nrf2 inhibitor ML385 was supplied by Selleck (Houston, TX, USA). The cells (5 × 10^5^ cells/ml) were grown in 6-well plates. After pretreatment with 4 μg/ml ME for 8 h, 10 ng/ml human transforming growth factor (TGF-β1; Cell Signaling Technology, MA, USA) was used to stimulate CCD-18Co cells for 12 h. Through this, the expression of fibrosis-associated molecules was stimulated. Lipopolysaccharide (LPS) was purchased from Sigma (Suzhou Heyi Biotech Co. Ltd., Suzhou, China.). Cells were seeded in 6-well plate at a density of 5 × 10^5^ cells/ml. Furthermore, LPS (1 μg/ml) and ME (4 μg/μl) were added to a 6-well plate followed by further incubation for 4 h at 37°C. Then total RNA was extracted and quantitative real-time PCR analysis was conducted.

### Cell Viability Measurements by CCK-8 Assay

CCD-18Co cells (1 × 104 cells/well) were grown in a 96-well microplate and maintained in a humidified 5% CO2 atmosphere at 37°C for 24 h. The cells were exposed to different concentrations of ME for 24 h. Cell Counting Kit-8 (CCK-8; Vazyme Biotech, China) was used to measure cell viability. Then, CCK8 was added to each well immediately after a 4-h incubation at 37°C. The absorbance values were read at 450 nm (A450) using a microplate reader. To calculate the mean values, the experiment was performed for three times.

### Small Interfering RNA Transfection and Treatments

The Nrf2 small interfering RNA (siRNA) target sequence (5′-GCCUGUAAGUCCUGGUCAUTT-3′) and negative control siRNA sequence (5′-AUGACCAGGACUUACAGGCTT-3′) were supplied by Ribo Biotech (Guangzhou, China). After 4-h of starvation, Lipofectamine 2000 (Invitrogen, USA) was used to transfect siRNA into the cells in compliance with the manufacturer’s protocol. Following transfection for 24 h, the cells were treated for 8 h with or without ME, and then exposed to 10 ng/ml TGF-β1.

### Western Blotting

RIPA lysis buffer was used to isolate proteins from the cells or colon tissues (Beyotime Biotechnology, Shanghai, China). The buffer contained 1 mM Pierce™ phosphatase inhibitor and 0.1% Halt™ protease inhibitor cocktail. These inhibitors were procured from Thermo Fisher Scientific, Shanghai, China. The centrifugation was performed afterwards (20 min, 13000 rpm, 4°C) and the supernatants were collected. BCA protein assay kit (Pierce Biotechnology, Rockford, Illinois, USA) was employed to determine the protein concentrations. After mixing with 5x SDS-PAGE sample buffer, the same amounts of proteins (30 μg) were separated on a 10% SDS-PAGE gel, and then transferred onto PVDF membranes (Merck Millipore, USA). Then, the samples were blocked in a 5% BSA buffer for 90 min before incubating the membranes with the corresponding primary antibodies (Abcam, CA, USA; Nrf2, HO-1, α-SMA, Collagen I, Collagen III, phosphorylated SMAD2, phosphorylated SMAD3, TIMP1, TIMP3, MMP3, TNF-α, IL-6, IL-1β, pIκB and NFκB p65 (phospho S536)) overnight at 4°C. The samples were then rinsed with TBST for three times, followed by incubation with a secondary antibody (Biogot technology Co. Ltd., Nanjing, Jiangsu, China) for 90 min at ambient temperature. To visualize the protein bands, Immobilon Western HRP substrate (Beyotime Biotechnology, Shanghai, China) was used to incubate the membranes. All protein bands were normalized to GAPDH.

### RNA Extraction and Quantitative Real-Time PCR Analysis

To extract total RNA, FastPure Cell/Tissue Total RNA Isolation Kit (Vayzme, Nanjing, Jiangsu, China) was used following the manufacturer’s protocol. To synthesize complementary DNA (cDNA), 500 ng of total RNA was subjected to reverse-transcription using the PrimeScript™ RT Master Mix (Takara, Beijing, China). QRT-PCR analyses of duplicate samples were performed on QuantStudio 5 (Thermo Fisher Scientific, Shanghai, China) using Applied Biosystems Power SYBR Green PCR Master Mix (Thermo Fisher Scientific, Shanghai, China). The relative expression of target genes was normalized to GAPDH. **Table 1** lists the primer sequences of the target genes. The critical threshold cycle (CT) value and the relative mRNA concentrations (E =2^-ΔΔCt^) were measured for each reaction. Samples were performed in triplicate and every experiment was performed at least three times. The transcription levels of Nrf2, HO-1, α-SMA, COL1 α 1, MMP3, TIMP1, IL-6, TNF-α, IL-1β, IL-10, iNOS, and Arg-1 were determined by qPCR assay.

**Table 1:** Sequences of primers designed for RT-qPCR.

Gene	Source	Forward sequence	Reverse sequence
Nrf2	Human	CATCCAGTCAGAAACCAGTGG	GCAGTCATCAAAGTACAAAGCAT
HO-1	Human	GGGTGATAGAAGAGGCCAAGACT	CAGCTCCTGCAACTCCTCAAA
α-SMA	Human	TGTGGCTATCCAGGCGGTGC	TCTCGGCCAGCCAGATCCAGAC
COL1α1	Human	GAGGGCCAAGACGAAGACATC	CAGATCACGTCATCGCACAAC
MMP3	Human	TGCTTTGTCCTTTGATGCTG	GATTTTCCTCACGGTTGGAG
TIMP1	Human	CTGTTGTTGCTGTGGCTGAT	GTTGTGGGACCTGTGGAAGT
GAPDH	Human	GCACCGTCAAGGCTGAGAAC	TGGTGAAGACGCCAGTGGA
Nrf2	Mouse	AACAACGCCCTAAAGCA	TGGATTCACATAGGAGC
HO-1	Mouse	CACGTATACCCGCTACCT	CCAGTTTCATTCGAGCA
α-SMA	Mouse	ATGAAGCCCAGAGCAAGAGA	TCCAGAGTCCAGCACAATACC
TGF-β1	Mouse	TGACGTCACTGGAGTTGTACGG	GGTTCATGTCATGGATGGTGC
COL1α1	Mouse	GAACCCCAAGGAAAAGAAGC	TGCTGTAGGTGAAGCGACTG
TIMP1	Mouse	TCCCCAGAAATCAACGAGAC	ACCCCACAGCCAGCACTAT
TNF-α	Mouse	TCTCCAGCCACCAGCCCTCTAA	TGGCCATGGTAGGAGAAACAGG
IL-6	Mouse	ACAAAGCCAGAGTCCTTCAGAG	GGCAGAGGGGTTGACTT
IL-1β IL-10 Arg-1 iNOS	Mouse Mouse Mouse Mouse	GGATGATGATGATAACCTGC AGCCTTATCGGAAATGATCCAGT CTCCAAGCCAAAGTCCTTAGAG GTTCTCAGCCCAACAATACAAGA	CATGGAGAATATCACTTGTTGG GGCCTTGTAGACACCTTGGT AGGAGCTGTCATTAGGGACATC GTGGACGGGTCGATGTCAC
GAPDH	Mouse	AGGTCGTGTGAGGGATTG	TTAGTAGTAGTAGGGGGGGTCA

### Measurement of Tissue Collagen Content

Hydroxyproline was used to estimate the content of tissue collagen according to the Woessner’s method as described previously (26–28). The hydroxyproline assay kit used in this study was supplied by Nanjing Jiancheng Bioengineering Institute (Nanjing, China).

### Immunohistochemical and Immunofluorescence Analyses

After fixing in 4% paraformaldehyde and embedding in paraffin, the colon tissue was dewaxed in dimethylbenzene, dehydrated in a gradient of ethanol, and then washed with deionized water. After blocking with BSA (5%) in PBS for 30 min, the sections were incubated overnight at 4°C with goat polyclonal collagen I antibody (1:100; Santa Cruz Biotechnology, CA, USA), rinsed with PBS for 15 min, and incubated again with secondary antibody for 30 min at ambient temperature. After staining with DAB, the sections were counter-stained with hematoxylin.

To perform immunofluorescence staining, the sections were prepared following the same steps as above until antigen retrieval. The sections were then blocked with 1% BSA for 1 h, and incubated overnight at 4°C with primary antibodies. Subsequently, immunofluorescence staining was performed with Alexa Flour 488 or 594-conjugated secondary antibody. After incubation with secondary antibody, DAPI solution was used to stain the nuclei under dark conditions. Finally, the stained nuclei were examined using a Leica DMIRE2 confocal laser scanning microscope.

### Histopathological Assessment of Possible Toxic Effects

Blood specimen was collected from each mouse after scarification The shape, size, position and color of the internal organs were monitored and recorded. Ten percent formalin was used to preserve the spleens, livers, kidneys and colons. Afterwards, the tissues were paraffin-embedded and sectioned, followed by H&E staining.

### Measurement of Gut Microbiota

The stool was sampled from the mouse colon and kept at −80°C for further use. DNA extraction was conducted on 0.18-0.22 kg of stool samples using the TIANamp Stool DNA Kit (Qiagen, Germany) in compliance with the manufacturer’s protocol. A NanoDrop spectrophotometer (Thermo Scientific, USA) was used to measure the quality and yield of the DNA extracts. The low-quality PUE group sample (PUE4) was excluded for further analysis. For amplification of the V4 hypervariable region of the 16S rRNA gene, 806R (5′-GGACTACHVGGGTWTCTAAT-3′) and primers 515F (5′-GTGCCAGCMGCCGCGGTAA-3′) were used (29). To perform high-throughput sequencing, an Illumina MiSeq platform (Novogene Corporation) was used based on the PE250 strategy (paired-end 250 bp sequences).

### Assessment of Biochemical Markers

After repeated “cycles”, fasted blood samples were collected from the sacrificed animals for clinical chemistry assessment. Blood was collected *via* cardiac puncture. The blood was placed in a plane test tube for an hour. To obtain serum, the blood was centrifuged for 10 min with an electrical centrifuge at 3500 rpm. Then serum was separately collected by micropipette and kept in a vial. Finally, the activities of hepatic enzymes such as alanine transaminase (ALT) and aspartate transaminase (AST) were quantified spectrophotometrically using commercially available kits (Abcam, CA, USA) indicating hepatic functions were recorded: alanine aminotransferase (ALT), aspartate aminotransferase (AST). ALT and AST were considered as the primary outcome measures to test the efficacy of the intervention.

### Statistical analysis

The data were presented as mean ± standard deviation (SD) based on at least three replicates. To examine the difference between the two groups, Student’s t-test was carried out. The differences among three or more groups were compared using ANOVA (P ≤ 0.05). Student’s t-test was used with 2-way ANOVA for inter group comparisons. All analyses were conducted in GraphPad Prism ver. 6.07 (San Diego, CA, USA).

## Results

### ME Exerts Protective Effects on Body Weight, Macroscopic Appearance, Colon Length, and DAI in DSS-Induced Chronic Colitis

To better elucidate the potential therapeutic effects of ME in chronic colitis, we constructed a mouse fibrosis model induced by repeated “cycles” of DSS exposure (**Figure 1A**), which is widely utilized to study intestinal fibrosis of IBD (30, 31). The stool consistency, body weight loss, and rectal bleeding are all major symptoms of DSS-induced colitis. The results showed that body weight loss in the mice exposed to DSS started from Day 5. As similar to mesalazine, which is commonly prescribed for treating IBD, ME could markedly ameliorate the loss of body weight in DSS-exposed mice (**Figure 1B**). Besides, a significant decrease in the length of colon was observed in the colitis mice when compared with the control mice (**Figures 1C, D**), indicating that severe intestinal inflammation and fibrosis are induced by DSS. However, treatment with ME significantly reversed colon shortening in DSS-exposed mice. It was also found that DSS led to spleen enlargements accompanied by inflammatory changes (**Figures 1E, G**). However, treatment with ME or mesalazine was able to suppress spleen enlargement. More importantly, ME and mesalazine had relatively similar pharmacological effects on DSS-induced chronic colitis. In addition, the DAI score (an indicator of colitis severity) was remarkably higher in DSS-exposed mice than in those fed with water only; and treatment with ME clearly reduced the score (**Figure 1F**). To determine the mechanisms underlying the effects of ME on inflammation-associated intestinal fibrosis, an Nrf2 inhibitor (ML385) was used in the subsequent analysis. According to previous reports (32, 33), 30 mg/kg of ML385 was given intraperitoneally 1 h before Nrf2 inhibition by ME. The results showed that the beneficial effects of ME on DSS-induced colitis were reduced by ML385 pretreatment (**Figures 1B–F**), indicating that ME could alleviate the severity of DSS-induced chronic colitis *via* Nrf2 pathway activation.

### ME Reduces the Inflammation Caused by DSS-Induced Chronic Colitis *via* Nrf2 Pathway Activation

**Figure 2:**
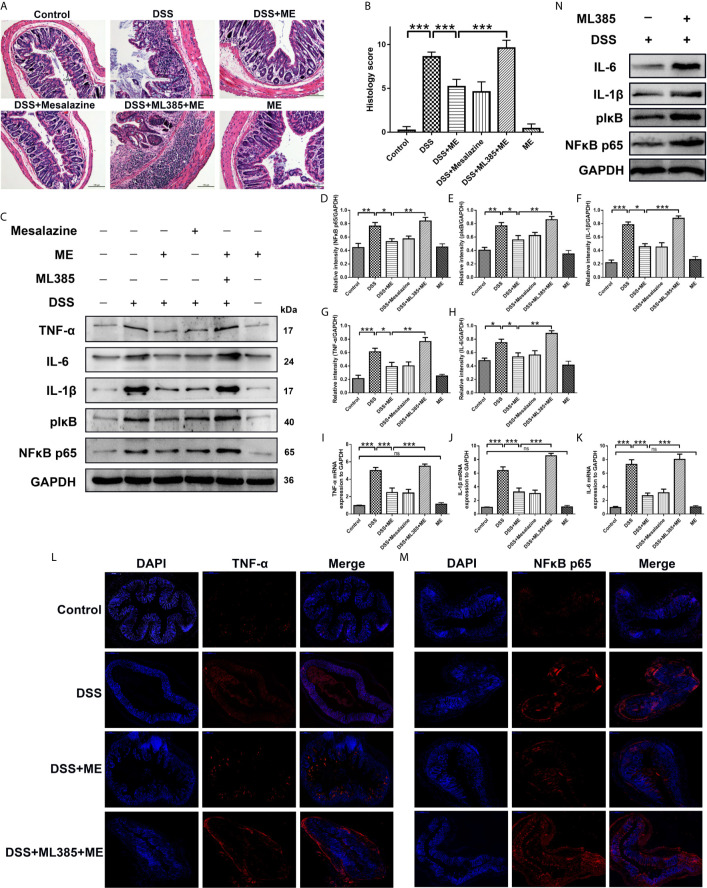
ME alleviates DSS-induced colon damage and exhibits anti-inflammatory effects on DSS-induced colitis *via* Nrf2 activation. Mice were allocated to six groups (n=8 per group). After treatment, colons were removed, sectioned and then stained with H&E. The representative images of the stained sections **(A)** and histology scores **(B)**. Scale bar = 100 μm. CoEp, columnar epithelium; GoCe, goblet cell; InCe, inflammatory cell; InCr, intestinal crypt. The protein expression of pIκB, IL-1β, IL-6, NFκB p65 (phospho S536) and TNF-α in colonic tissues was examined using Western blot analysis. Representative data are demonstrated **(C)** and the relative protein intensities of pIκB, IL-6, IL-1β, NFκB p65 (phospho S536) and TNF-α were normalized to GAPDH **(D–H)**. The mRNA levels of TNF-α, IL-1β and IL-6 in colonic tissues **(I–K)**. To reveal different molecules (red), immunostaining was conducted on the colon tissue section **(L, M)**. To counterstain the slides, DAPI (blue), an inverted fluorescence microscope was used for imaging (×200). Data are presented as mean ± SD. ^∗^P < 0.05, ^∗∗^P < 0.01, and ^∗∗∗^P < 0.001; ns, no significance. **(N)** The protein expression of pIκB, IL-1β, IL-6 and NFκB p65 (phospho S536) in colonic tissues was examined using Western blot analysis.

The specialized colonocytes found in the intestinal walls have numerous functions and act as the physical and chemical defense against foreign invaders (34). The results of histopathological analyses also supported the effects of ME on DSS-induced colitis. Notably, DSS caused considerable epithelial damages, infiltration of dense inflammatory cells, loss of goblet cells and crypts, and mucosal ulcers (35–37). Interestingly, ME exhibited a considerable preventive effect on colonic tissue injury (**Figure 2A**) and reduced the histological scores in DSS-induced colitis (**Figure 2B**). This indicates that ME can protect the tissue against intestinal damage in chronic colitis mice. Moreover, the colitic mice treated with ME had no apparent histological changes (**Figures 2A, B**), and those pretreated with ML385 displayed no protective effects (**Figures 2A, B**). These results imply a potent negative regulatory role played by ME to suppress inflammatory responses. To explore the mechanism underlying the protective effect of ME, the signaling pathways associated with intestinal inflammation were investigated (**Figures 2C–M**). Nrf2 is a main regulator of detoxification, which has been shown to activate the transcription of genes encoding for antioxidant enzymes. Thus, the role of Nrf2 in mediating the protective effects of ME on DSS-induced colitis was examined. Compared with the control mice, DSS-exposed mice had markedly higher expression levels of pIκB, IL-6, IL-1β, NFκB p65 (phospho S536) and TNF-α (**Figures 2C–M**). In contrast, the protein and mRNA levels of these inflammatory cytokines were remarkably decreased after ME treatment or mesalazine (**Figures 2C–M**). It was also found that ML385 (Nrf2 inhibitor) reduced the antagonizing effects of ME on inflammatory reactions. Collectively, these results indicate that ME ameliorates histological damages in chronic colitis mice *via* activation of Nrf2 pathway.

### ME Improves the Histological Parameters of Chronic Colitis-Associated Intestinal Fibrosis

**Figure 3:**
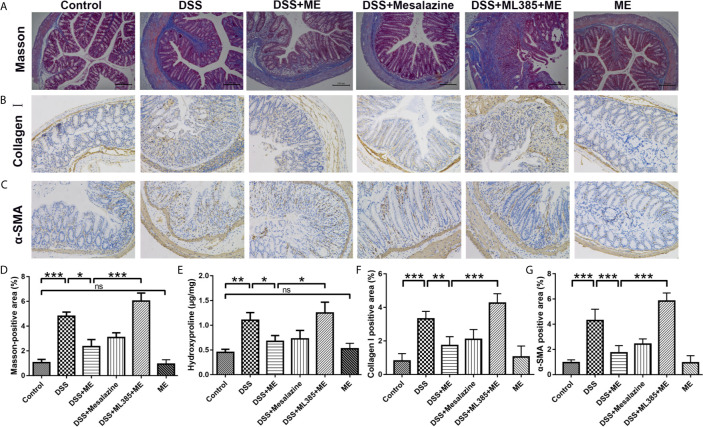
ME inhibits the high levels of fibrosis markers in histological assays. **(A)** Representative image of the Masson trichrome-stained colon sections in the six groups: ECM deposition (blue area). **(B)** Representative immunohistochemical staining for α-SMA in colon sections and quantitative analysis. **(C)** Representative immunohistochemical staining for collagen I in colon sections and quantitative analysis. **(D)** Quantitative analysis of the masson-positive area. **(E)** Hydroxyproline assay of colon tissues from the mice in six different groups. Scale bar = 200 μm. **(F)** Quantitative analysis of the collagen I-positive area. **(G)** Quantitative analysis of the α-SMA-positive area. Data are presented as mean ± SD. ^∗^P < 0.05, ^∗∗^P < 0.01, and ^∗∗∗^P < 0.001; ns, no significance.

Severe colitis caused by DSS can lead to acute intestinal inflammation during the progression and deterioration of IBD. The complications include abnormal epithelial structure, thicker gut wall, persistent collagen deposition, and mononuclear cell infiltration. In addition, both α-SMA and collagen I were the prominent biomarkers for assessing intestinal fibrosis (38–40). An intestinal fibrosis model was established (**Figure 1A**) to study the effects of ME on chronic colitis complications. Masson trichrome staining and immunohistochemical assay were carried out to visualize the fibrosis-related mucosal and submucosal collagen accumulation in chronic colitis mice. The results showed that ME exerted protective effects against fibrosis-associated collagen deposition (**Figures 3A–G**). To further evaluate the severity of colon fibrosis, the levels of hydroxyproline in colon tissue were determined (**Figure 3E**). Notably, the concentration of hydroxyproline in DSS exposure group was obviously increased compared to the control group. However, ME treatment markedly decreased its level. Furthermore, in the presence of ML385, the protective effects of ME on mice with inflammation-associated intestinal fibrosis were abolished (**Figure 3**). These histological parameters also support that ME exerts its beneficial effects on chronic colitis-associated intestinal fibrosis *via* Nrf2 pathway.

### ME Attenuates Intestinal Fibrosis in DSS-Induced Chronic Colitis by Activating Nrf2-Mediated Pathway

**Figure 4:**
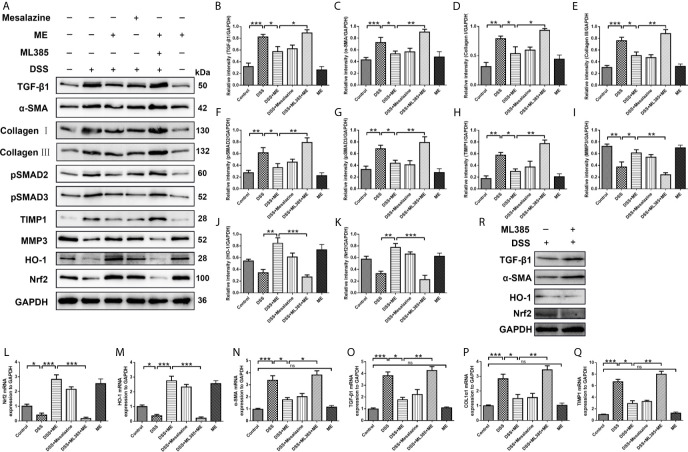
ME suppresses inflammation-associated intestinal fibrosis by suppressing TGF-β1/SMADs pathway *via* upregulating Nrf2 expression. Mice were allocated to six groups (n=8 per group). **(A)** Effects of ME on TGF-β1/SMADs signaling pathways and Nrf2 pathway in DSS-induced chronic colitis. Representative data are shown in **(A)**. The relative protein intensities **(B–K)** were normalized to GAPDH. **(L–Q)** mRNA expression levels of the fibrosis markers were detected and normalized to GAPDH. **(R)** The inhibitor ML385 aggravated the colitis. The data are presented as mean ± SD. ^∗^P < 0.05, ^∗∗^P < 0.01, and ^∗∗∗^P < 0.001; ns, no significance.

Fibrosis involves the secretion of a series of fibrosis-related maker proteins, including collagen I, α-smooth muscle actin (α-SMA), MMP3, ECM-degrading protein and TIMP-1, which exhibit a direct role in the fibrogenesis. However, it remains unknown whether ME can affect the activation of fibroblasts and expression of fibrotic markers. In our previous work, we demonstrated that ME could reduce oxidative stress and alleviate inflammation in experimental colitis through Nrf2 pathway activation, thereby leading to a general enhancement in both histological and macroscopic parameters (17, 41). In this study, the possible involvement of Nrf2 during the treatment of ME for chronic colitis-associated intestinal fibrosis was also examined. The results showed that the expression levels of profibrotic factors in TGF-β1/SMADs signaling pathway, such as TGF-β1, α-SMA, Collagen I, Collagen III, pSMAD2, pSMAD3 and TIMP1, were remarkably higher in DSS-exposed mice than in control mice (**Figures 4A–K**). After treatment with ME or mesalazine, there was a significant reverse in the expression levels of these proteins (**Figures 4A–K**). However, the expression of MMP3 in mice with DSS-induced colitis was markedly upregulated following ME treatment (**Figures 4A, I**). In addition, the protein expression levels of HO-1 and Nrf2 in DSS-exposed mice were significantly reduced due to chronic colitis (**Figures 4A, J, K**). Consistently, ME and mesalazine restored or even further enhanced the expression levels of Nrf2 and HO-1 (**Figures 4A, J, K**). Besides, ML385 (Nrf2 inhibitor) suppressed the antagonizing effects of ME on intestinal fibrosis caused by chronic colitis. To further determine the roles of ME in chronic colitis, qRT-PCR was used to detect the mRNA levels of fibrosis makers and Nrf2 in the colon tissues (**Figures 4L–Q**). Again, the results were similar to those of Western blotting. The colitic mice treated with ME still exerted protective effects after ML385 pretreatment (**Figures 4L–Q**). When we used the inhibitor ML385 directly, the colitis got worse (**Figure 4R**). These findings indicate that ME has beneficial effects on inflammation-associated intestinal fibrosis by suppressing TGF-β1/SMADs pathway *via* upregulating Nrf2 expression.

### ME Inhibits TGF-β1/SMADs Signaling Pathway in a Dose-Dependent Manner in CCD-18Co Cells

**Figure 5:**
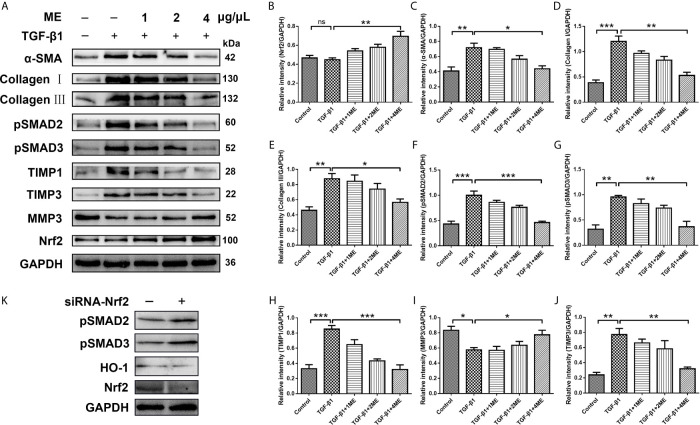
ME suppresses the expression levels of fibrosis markers in TGF-β1-stimulated CCD-18Co cells. **(A)** TGF-β1-stimulated CCD-18Co cells were treated with ME for 8 h at different doses, and the protein levels of Nrf2 and fibrosis markers were detected and normalized to GAPDH. **(B–J)** Western blot bands were analyzed by densitometry. **(K)** SMADs signaling were detected by silencing Nrf2 without exogenous TGF-β. Data are presented as mean ± SD. ^∗^P < 0.05, ^∗∗^P < 0.01, and ^∗∗∗^P < 0.001; ns, no significance.

CCD-18Co fibroblasts can be differentiated into ECM-producing myofibroblasts *via* activation of TGF-β1. To investigate the potential antifibrotic effect of ME, Western blotting was used to assess the expression of α-SMA, Collagen I, Collagen III, pSMAD2, pSMAD3, TIMP1, TIMP3 and MMP3 induced by TGF-β1 in CCD-18Co cells treated with or without different concentrations of ME. It was found that ME increased the protein levels of Nrf2 in a dose-dependent fashion (**Figure 5A**). Moreover, our results indicated that, after TGF-β1 stimulation, the CCD-18Co cells displayed higher expression levels of fibrosis markers, including α-SMA, Collagen I, Collagen III, pSMAD2, pSMAD3, TIMP1 and TIMP3 (**Figure 5A**). However, treatment with ME markedly suppressed the TGF-β1-induced expression levels of the fibrosis markers in a dose-dependent fashion (**Figure 5**). Furthermore, ME treatment led to a markedly higher expression of MMP3 when compared to the untreated TGF-β1-induced CCD-18Co cells (**Figure 5I**). Otherwise, our data showed that SMADs signaling were activated by silencing Nrf2 without exogenous TGF-β (**Figure 5K**).

### TGF-β1-Induced Differentiation of CCD-18Co Cells and LPS-Challenged RAW264.7 Cells Are Prevented by ME Treatment *via* Upregulating Nrf2 Expression

**Figure 6:**
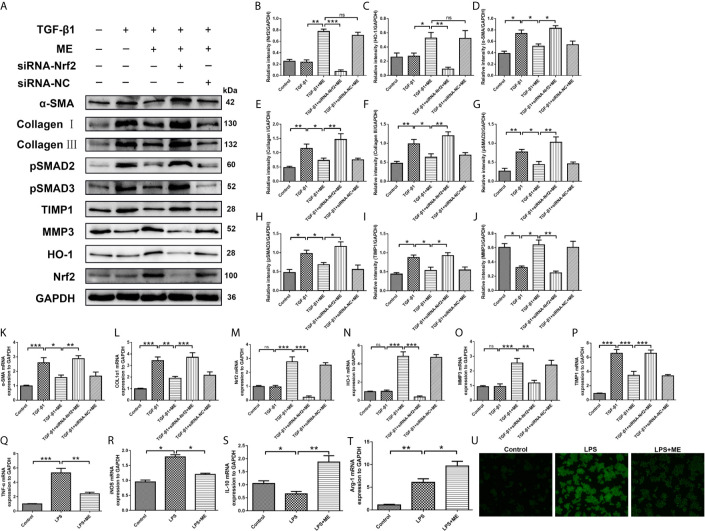
ME inhibits TGF-β1-induced differentiation of CCD-18Co cells *via* upregulating Nrf2 expression. **(A)** Cells were treated with a combination of TGF-β1, ME, siRNA-Nrf2 or siRNA-NC, and the protein levels of Nrf2, HO-1, α-SMA, Collagen I, Collagen III, pSMAD2, pSMAD3, TIMP1 and MMP3 were detected and normalized to GAPDH. Representative data are demonstrated. **(B–J)** Western blot bands displayed in **(A)** were evaluated using densitometry. **(K–P)** mRNA expression levels of the fibrosis markers were detected and normalized to GAPDH. **(Q–T)** mRNA expression levels of macrophage markers were detected and normalized to GAPDH. **(U)** RAW 264.7 cells were stimulated with LPS. Green fluorescence represented the generated ROS. Data are presented as mean ± SD. ^∗^P < 0.05, ^∗∗^P < 0.01, and ^∗∗∗^P < 0.001; ns, no significance.

During IBD occurrence, the continuous dysregulation of colon damage-healing program can induce an abnormal ECM protein deposition and progressive fibrosis. The main cellular mechanism of intestinal fibrosis in this process is fibroblast activation (42), and the key intestinal fibrosis markers are α-SMA and collagen (40). In addition to inflammation and oxidative stress, TGF-β is also involved in intestinal fibrosis, both *via* a canonical interaction with SMADs as well as a complex network with other profibrotic and antifibrotic molecules (6, 10, 43). There have been numerous studies on Nrf2, especially on its interaction with TGF-β and its role in regulating the fibrogenesis process of various organs (e.g., the bowel) (44–48). To elucidate the key mechanisms underlying the anti-fibrotic effects of ME, the relationship between ME treatment and colon fibroblast activation was examined. As shown in **Figure 6**, the protein levels of Nrf2, HO-1, α-SMA, Collagen I, Collagen III, TIMP1 and TIMP3 were markedly increased in fibrosis mice compared with control mice (**Figure 6A**). However, treatment with ME remarkably downregulated the expression levels of these intestinal fibrosis markers in fibrosis mice. As the main profibrotic transcription factors, SMAD2 and SMAD3 were activated in DSS-induced chronic colitis, and the increased phosphorylation levels of SMAD2 and SMAD3 were reversed by ME treatment (**Figures 6A, G, H**). Besides, treatment with ME noticeably increased the expression level of MMP3 expression in TGF-β1-induced CCD-18Co cells (**Figures 6A, J**). Nevertheless, transfection with Nrf2-siRNA markedly upregulated the TGF-β1-induced protein levels of phosphorylated SMAD2, phosphorylated SMAD3, α-SMA, Collagen I, Collagen III and TIMP1 compared with the negative control (**Figures 6A–I**). Moreover, we found that the antifibrotic effects of ME in TGF-β1-stimulated CCD-18Co cells were offset by silencing Nrf2 (**Figures 6A, B**). Again, the results of qRT-PCR analysis (**Figures 6K–P**) were similar to those of Western blotting. Thus, we demonstrated that ME directly inhibited the activated TGF-β1/SMADs signaling pathway *via* upregulating Nrf2 expression in intestinal fibrosis. The largest population of macrophages in the body resides in the gastrointestinal tract, which is an important component of protective immunity (49). M1 macrophages can release inflammatory cytokines to induce an inflammatory response. However, M2 macrophages can secrete interleukin-10 (IL-10) to exert anti-inflammatory effects (50). To access the direct effect of ME on macrophages, a commonly used macrophage cell line RAW264.7 was adopted. Importantly, ME decreased the mRNA levels of M1 macrophage markers iNOS and TNF-α, and increased M2 markers IL-10 and Arg-1 in LPS-challenged RAW264.7 cells (**Figures 6Q–T**), suggesting a function of ME in polarizing macrophages from a pro-inflammation to an anti-inflammation phenotype. Meanwhile, we recorded fluorescence photographs of the produced ROS stimulated by LPS and scavenging by ME in RAW 264.7 cells (**Figure 6U**). The endogenous ROS-scavenging abilities of ME are evident in the decreased intensity of the green fluorescence/ROS signal compared with that of the LPS-treated cells (**Figure 6U**).

### *In Vitro* and *In Vivo* of Biocompatibility of ME

**Figure 7:**
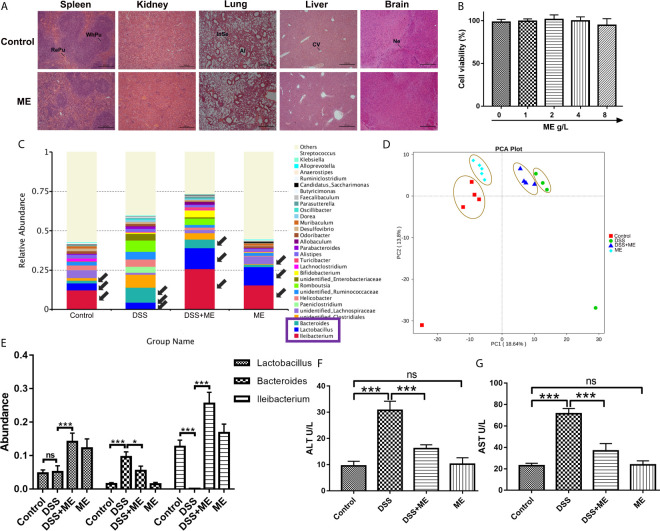
ME exhibits excellent biocompatibility both *in vivo* and *in vitro*. **(A)** The brain, liver, lung, kidney and spleen were sectioned, followed by H&E staining. Scale bar = 500 μm. Al, alveolus; CV, central vein; InSe, interalveolar septum; Ne, neuron; RePu, red pulp; WhPu, white pulp. **(B)** *In vitro* cytotoxicity of different concentrations of ME in CCD-18Co cells following 24 h of incubation, as revealed by CCK-8 assay. **(C)** Taxonomic composition at the genus level. **(D)** Mice were allocated to four groups (n=4-5 per group). PCA of the tested samples *via* bacterial 16S rRNA gene sequencing. **(E)** Abundance of *Lactobacillus*, *Bacteroidetes*, and *Ileibacterium* species. **(F, G)** Enzyme activities of serum ALT and AST were analyzed in mouse model. Data are presented as mean ± SD. *P < 0.05, **P < 0.01, and ***P < 0.001; ns, no significance.

The potential toxicity of ME was examined both *in vivo* and *in vitro*. No obvious clinical sign of toxicity was found in the mice exposed to ME. The microscopic examination indicated no discernible treatment-related abnormalities after ME treatment (**Figure 7A**). The histopathological assessment of the main organs of mice indicated that no clinically significant abnormality observed in the brain, liver, lung, kidney, spleen (**Figure 7A**) or colon (**Figure 2A**) compared with untreated controls (**Figure 7A**). ME treatment exhibited no overt toxicity regarding the loss of body weight and abnormal behavioral patterns (**Figure 1B**). In addition, no significant differences in clinical features (e.g., food or water intake, diarrhea, or other symptoms) were observed between ME treatment and control groups (**Figure 1**), and all mice were survived throughout the experiment. The results of CCK-8 assay showed that ME treatment (up to 8 g/L) for 24 h had no significant effect on the viability of CCD-18Co cells, suggesting the minor cytotoxic effect of this extract (**Figure 7B**). In addition, the development of IBD involves complex interactions between genetic variation, environmental factors, gut microbiota and the immune system. The occurrence and development of colitis is closely related to the disturbance of gut microbiota. To determine whether gut microbiota can affect DSS-induced chronic colitis mice, the abundance and composition of the intestinal microflora were analyzed through bacterial 16S rRNA gene (V4 region) sequencing. As shown in **Figures 7C–E**, ME notably restored the diversity of the gut microbiota in DSS-induced chronic colitis mice at the genus level. Furthermore, ME treatment affected the ratio of Lactobacillus and downregulated the ratio of Bacteroides (**Figures 7C, E**). Normal mice administrated with ME showed no gross abnormality compared with control group (**Figures 7D, E**). The presence of tissue damage in the liver can be evaluated by measuring serum level of enzymes like alanine aminotransferase (ALT) and aspartate aminotransferase (AST). Tissue damage results in the release of extra AST and ALT to the blood circulation and thus, the blood level of such enzymes is increased. Therefore, these enzymes are important biomarkers of liver damage. ALT is the most sensitive (51). In consistent, ME decreased the serum levels of ALT and aspartate aminotransferase AST, indicating a reduced liver damage in mouse models (**Figures 7F, G**).

## Discussion

IBD includes a range of diseases, such as UC and CD, which may result in different complications of intestinal fibrosis, oxidative stress and cancer. These complications are mostly manageable by surgical operation (52). In the progression and deterioration of IBD, acute colitis caused by DSS definitely progressed to severe chronic colon inflammation with irregular epithelial structure, thickening of gut wall, numerous infiltrates of mononuclear cells, and persistent deposits of collagen. α-SMA and collagen I were considered as the main intestinal fibrosis markers. Intestinal fibrosis is mainly manifested as an excessive accumulation of ECM proteins through myofibroblast activation (10, 53). As fibrosis is often initiated along with intestinal inflammation, colon fibrosis is considered as an outcome of dysregulated colon damage-healing and chronic intestinal inflammation in IBD (7). Under normal conditions, the injured tissues are healed through the colon recovery program; but when the damage is uncontrolled, it may lead to the development of chronic intestinal inflammation together with continuous injury and repair events, and ultimately colon fibrosis (10). Several studies have demonstrated that inflammatory response is required for the initiation and progression of colon fibrosis (54, 55). For instance, the currently available anti-inflammatory therapies fail to reverse or prevent intestinal fibrosis progression, especially when ECM proteins are excessively accumulated. Therefore, it is generally believed that the progression of IBD to colon fibrosis involves some detrimental factors that cause damage to the colon followed by direct inflammatory response. The repair of the damaged tissues is often accompanied by intestinal inflammation. When the physiological healing process compensates the damaged tissues, no further action is needed; otherwise, abnormal tissue repair (or known as fibroplasia) will be activated. Ultimately, ECM proteins are deposited into normal colon tissues, which in turn results in the formation of colon fibrosis (6, 55, 56). Aminosalicylates, corticosteroids, immunosuppressors and other biological agents are commonly used to ameliorate the clinical symptoms and inflammation caused by IBD. However, some adverse effects have been reported on these drugs, and thus, more effective, safer and targeted drugs are urgently needed for IBD treatment. A previous study has shown that a cecropin-like peptide obtained from maggots could exhibit considerable antimicrobial activities and anti-inflammatory responses against Gram-negative bacteria (57). Thus, the potential effects of ME on DSS-induced IBD were explored in this study. Our previous works (17, 41) have verified the protective effects of ME on acute inflammation and oxidative stress. However, the actual molecular mechanism responsible for ME functions in chronic inflammation-associated intestinal fibrosis remains largely unknown. The present study clearly demonstrated that ME attenuates inflammation-associated intestinal fibrosis by suppressing TGF-β1/SMADs pathway *via* upregulating Nrf2 expression.

In this study, we established a mouse model of chronic colitis to determine the beneficial roles of ME treatment. One of the well-known mouse models is DSS-induced chronic colitis that has several signs and symptoms in common with human IBD (e.g., emaciation, diarrhea, body weight loss, mucosal ulceration, shorten colon length, melena, and inflammatory cell infiltration) (58). After the administration of repeated “cycles” of 2% DSS, the mice with DSS-induced colitis displayed a remarkably higher DAI score compared with the control group (**Figure 1**). In addition, the results showed that DSS-induced pathological varies in the colonic tissue samples caused by HE staining. The mice in DSS group demonstrated continuous irregularities in the colonic architecture such as glandular defects, crypt loss, mucosal ulceration and inflammatory cell infiltration (**Figure 2A**). Additionally, following DSS treatment, there was a significantly higher histological score (**Figure 2B**). Therefore, chronic colitis was successfully induced in C57BB/6 mice with only 2% of DSS. Treatment with ME alleviated the symptoms of DSS-induced chronic colitis and pathological alteration in the intestine. However, the actual mechanism of IBD is still unknown, the findings supported the protective effects of ME on inflammation-associated intestinal fibrosis. In addition, by activating Nrf2, ME exerted a direct suppressive effect on the upregulation of proinflammatory cytokines (**Figures 2C–M**). A redox-sensitive transcription factor is NFκB, which is responsible for innate immunity, inflammation, and maintenance of tissue integrity. It also controls the expression of many proinflammatory factors (e.g., COX-2, IL-1β, IL-6, TNF-α and other chemokines). These cytokines can cause immune dysfunction and local inflammation, thus leading to colonic mucosal injury (59). The levels of these inflammatory cytokines are markedly both in experimental colitis (60) and UC patients (61). Our results showed the expression levels of pIκB, NFκB p65 (phospho S536), IL-1β, IL-6 and TNF-α were increased in colitis mice. Besides, ML385 alleviated the therapeutic effects of ME on inflammatory responses (**Figures 2C–M**). Thus, it is confirmed that ME could regulate chronic inflammation in colitis mice.

TGF-β1/SMADs signaling pathway is a critical mediator for intestinal fibrosis, and is the most potent cause of fibroblast activation (62, 63). A key element of ECM in the colon is collagen. Its deposition into the colon subepithelial layer was higher after the repeated administration of DSS. α-SMA is another key element of ECM and biomarkers for myofibroblast activation. Its expression was also higher following multiple administration of DSS (30). As reported in other studies, the overexpression of TGF-β causes colonic fibrosis, while the loss of SMAD3 can inhibit colon fibrosis (64, 65). Moreover, the balance between MMPs and TIMPs can be mediated by the deposition of ECM and intestinal fibrosis, which is often lost in the colon tissues of IBD patients (8). Thus, the effects of ME on TGF-β1/SMADs signaling pathway in chronic colitis model were examined. Our findings indicated that the overexpression of α-SMA, Collagen I, TGF-β1, Collagen III, pSMAD3, pSMAD2 and TIMP1 in DSS-induced colitis was downregulated by ME treatment (**Figure 4**). Moreover, the expression of MMP3 was decreased in DSS group and ME could markedly upregulate it (**Figures 4A, I**). Consistently, the antifibrotic effect of ME was also confirmed *in vivo*. The results showed that the suppressive effect of siRNA transfection on Nrf2 could lead to the overexpression of α-SMA, collagen I and other fibrosis-related indicators (**Figures 5**, **6**). Nrf2 acted as a protective agent against intestinal fibrosis based on both *in vitro* and *in vivo* experiments. It was noteworthy that there were no clinical signs of toxicity in the study organs, including brain, liver, lung, kidney, spleen and colon (**Figure 7A**). Furthermore, CCK-8 cell proliferation assays showed that ME treatment (up to 8 g/L) for 24 h had no obvious effect on the viability of CCD-18Co cells, indicating the minor cytotoxicity of ME (**Figure 7B**). In addition, the development of IBD involves complex interactions between genetic variation, environmental factors, gut microbiota and the immune system. The occurrence and development of colitis is closely related to the disturbance of gut microbiota (66–68). In order to explore the impact of ME intervention on the gut microbiota, we used 16S rRNA gene-based microbiota analysis. Similar to a previous study (69), DSS treatment perturbed the community composition of gut microbiota. According to our data, the composition of gut microbiota was perturbed and the richness of gut microbiota was lowered after DSS treatment. Interestingly, ME restored the diversity of gut microbiota in DSS-induced chronic colitis mice according to the detected genus level of tested samples as well as contributed to the ratio of Lactobacillus and downregulated the ratio of Bacteroides (**Figure 7C**). Normal mice administrated with ME showed no gross abnormality compared with control group (**Figure 7D**). In addition, our previous study had shown that ME exerted antioxidative activity (17), and its strong oxygen radical scavenging capacity may provide a survival advantage for Lactobacillus. ME could maintain the microbial balance of the gut by inhibiting pathogenic bacteria and stimulating the growth of beneficial bacteria, thus improving intestinal health. However, the mechanisms by which ME exerted probiotic effects and reshaping of the gut microbiota which benefits the host are still unclear. Nevertheless, ME exhibited good biocompatibility both *in vivo* and *in vitro*. As this has been the first time we explored the therapeutic efficacy of ME on gut microbiota in a mouse colitis model, certain limitations existed in our study. We preliminarily found that ME were beneficial to changes in gut microbial bacterial communities, thus briefly demonstrating that ME is non-toxic. We will further try to explore how ME intake affects the change of gut microbiota, and will focus on the metabolism of ME in mice.

As this has been the first time we explored the therapeutic efficacy of the maggot extracts in a mouse colitis model, certain limitations existed in our study. As the DSS model induces a severe form of colitis, we started the treatment from the beginning when DSS was given. Future studies may address the therapeutic effects of the maggot extracts on an already established disease. As our crude extracts are not suitable for intravenous or intradermal injection, the maggot extracts were delivered intragastrically to mice in the current study. We have not been able to compare the effect of different delivery routes on the efficacy of the treatment. Future experiments are warranted for evaluating the therapeutic potential of this protein in its purified, injectable form. Although general compounds such as amino acids, proteins, and nutrients are not expected to activate Nrf2, some minor compounds (maybe antimicrobial peptides) present in the maggot extracts can contribute to the Nrf2 activation. We may try to purify the maggot protein in subsequent experiments and then evaluate its potency in intravenous delivery by using Nrf2 KO mice (might be using broad analysis, e.g. RNAseq or Proteomics).

This study was the first to elucidate the mechanisms underlying the therapeutic efficacy of ME on chronic inflammation-associated intestinal fibrosis. However, there are inevitable limitations of our study. Considering that DSS can induce a severe form of colitis, ME treatment was performed immediately after DSS exposure. Besides, ME was administrated intragastrically as the crude extracts were not suitable for intradermal and intravenous administration. Moreover, this study did not compare the effects of different delivery routes on ME treatment efficacy. Therefore, future investigations are needed to verify the beneficial effects of ME on IBD patients and evaluate its therapeutic efficacy in pure and injectable dosage form.

## Conclusion

*In vivo* and *in vitro* data indicate ME exerts suppressive effect on inflammation-associated intestinal fibrosis by inhibiting TGF-β1/SMADs pathway *via* upregulating the expression of Nrf2, which results in a general enhancement of both histological and macroscopic parameters. The findings show that Nrf2 activators can be used to maintain the constant levels of this molecule in the early stages of IBD, thereby preventing the development and progression of cancer and fibrosis. ME exhibits excellent biocompatibility with an adequate safety profile, thus, it can be an alternative option to prevent and treat inflammation-associated intestinal fibrosis. Overall, the results highlight an infusive molecular mechanism of ME, which can serve as a potential therapeutic strategy for treating intestinal fibrosis.

## Data Availability Statement

The original contributions presented in the study are included in the article/supplementary material. Further inquiries can be directed to the corresponding author.

## Ethics Statement

The Institutional Animal Care and Use Committee of Nanjing University approved the animal care and *in vivo* experimental procedures based on the guidelines of institutional animal ethics (2019-124210-225A).

## Author Contributions

RW: Methodology, Investigation, Formal analysis, Writing - original draft. YZL: Methodology, Investigation, Formal analysis, Writing - original draft. DW and HW: Methodology, Investigation, Formal analysis, Writing - original draft, Conceptualization. TW: Investigation. YJW: Investigation. YZ: Investigation. YW: Conceptualization, Investigation, Formal analysis, Writing - original draft. All authors contributed to the article and approved the submitted version.

## Funding

This work was supported by the National Natural Science Foundation of China (grant numbers 81771539 and 81673430) and the Foundation of State Key Laboratory of Analytical Chemistry for Life Science (grant number 5431ZZXM1603). 

## Conflict of Interest

The authors declare that the research was conducted in the absence of any commercial or financial relationships that could be construed as a potential conflict of interest.

## Publisher’s Note

All claims expressed in this article are solely those of the authors and do not necessarily represent those of their affiliated organizations, or those of the publisher, the editors and the reviewers. Any product that may be evaluated in this article, or claim that may be made by its manufacturer, is not guaranteed or endorsed by the publisher.

## Abbreviations

IBD, Inflammatory bowel disease; UC, Ulcerative colitis; CD, Crohn’s disease; ME, Maggot extracts; Nrf2, NF-E2-related factor-2; DSS, Dextran sulfate sodium; TGF-β, Transforming growth factor-β; SMADs, small mothers against decapentaplegic proteins; IL, Interleukin; TNF, Tumor necrosis factor; HO, Heme oxygenase; NFκB, Nuclear factor κB; PBS, Phosphate-buffered saline; DAI, Disease activity index; IF, Immunofluorescence; MPO, Myeloperoxidase; ALT, alanine aminotransferase; AST, aspartate aminotransferase.
